# Supplementary material for: Canady Cold Helios Plasma Reduces Soft Tissue Sarcoma Viability by Inhibiting Proliferation, Disrupting Cell Cycle, and Inducing Apoptosis: A Preliminary Report
Source: Molecules. 2022 Jun 29;27(13):4168. doi: 10.3390/molecules27134168 (PMC9268132; doi:10.3390/molecules27134168)
Supplement: Supplementary file 1 [file molecules-27-04168-s001.zip › molecules-1766715-supplementary.pdf]

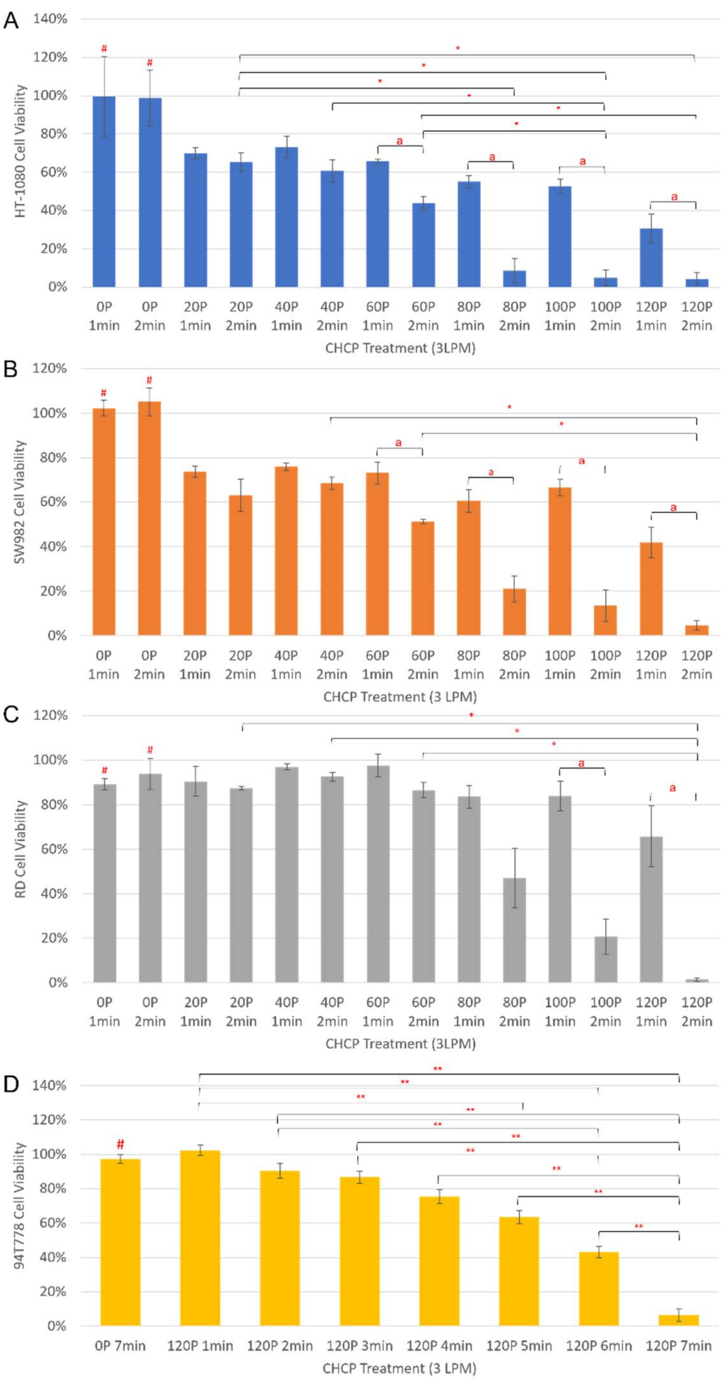

Figure S1. Bar graphs (taken from Figure 1 and Figure 2) showing the cell viability of **(A)** HT-1080, **(B)** SW982, **(C)** RD and **(D)** 94T778 cells 48-hour post-CHCP treatment compared to mock controls. Statistical significance for 1 min versus 2 min treatment (^{a} $p \leq 0.05$) was considered using a student t-test for **(A)** HT-1080, **(B)** SW982, and **(C)** RD cells. Statistical significance between different powers (20-120 P) (* $f \leq 0.0033$) were considered using a Post-Hoc test for **(A)** HT-1080, **(B)** SW982, and **(C)** RD cells. These tests did not find statistical difference for cells treated with a helium flow rate of 1 LPM, thus is not shown. Statistical significance between different treatment durations (1-7 min) (** $f \leq 0.002381$). Statistical significance was not considered (#) for Helium alone (0P) controls.
